# Large-scale mass wasting on small volcanic islands revealed by the study of Flores Island (Azores)

**DOI:** 10.1038/s41598-018-32253-0

**Published:** 2018-09-17

**Authors:** A. Hildenbrand, F. O. Marques, J. Catalão

**Affiliations:** 1 GEOPS, Univ. Paris-Sud, CNRS, Université Paris-Saclay, 91405 Orsay, France; 2 Universidade de Lisboa, Lisboa, Portugal; 3 Instituto Dom Luiz (IDL), Faculdade de Ciências, Universidade de Lisboa, Lisboa, Portugal

## Abstract

Small intra-plate volcanic islands (total height above seafloor <2500 m) have been considered gravitationally stable. Topographic, stratigraphic, structural and new K/Ar data show that the small island of Flores (Azores) is strongly asymmetric and made up of nested volcanic successions. Along the northwestern coastline, ca. 1.2 Ma lava flows are in lateral contact with a younger volcanic unit (ca. 0.7 Ma), reflecting the existence of a steep lateral discontinuity. From the general dip of the lava flows, their age and the arcuate geometry of the contact, we infer a major landslide that removed the western flank of the older volcano. Further inland, E-dipping lava flows at the summit of the island are ca. 1.3 Ma, suggesting another landslide structure that displaced the whole western half of the former volcanic edifice. Available offshore data show a large hummocky field west of Flores, here interpreted as voluminous debris-avalanche deposits. Unlike the eastern and central Azores islands, Flores sits on a relatively stable tectonic setting. Therefore, we propose that small-size volcanic islands can be sufficiently gravitationally unstable to experience recurrent episodes of large-scale mass wasting triggered by mechanisms other than tectonic earthquakes and thus represent an under-evaluated potential source of hazard and, therefore, risk.

## Introduction

Large-scale mass wasting (LSMW) on big oceanic islands (>5–6 km above the sea floor) can remove catastrophically large volumes of volcanic material and generate mega-tsunamis that may impact the whole surrounding shorelines, up to distances of several thousands of kilometers^1,2^. From on and offshore studies, giant landslides (>100 km^3^) have been recognized on large intra-plate volcanic islands, e.g. along the Hawaiian Ridge^3,4^, in Polynesia^5–7^, in the Canaries (e.g.^8–14^), in the Reunion Island (e.g.^15^) and in Cape Verde (e.g.^16^). Such large intra-plate volcanic edifices frequently reach a total height up to 10 000 m from the surrounding seafloor and are therefore the most gravitationally unstable. In contrast, small-size volcanic islands and seamounts (total height < 2500 m) are believed to lack LSMW (e.g.^17^).

**Figure 1:**
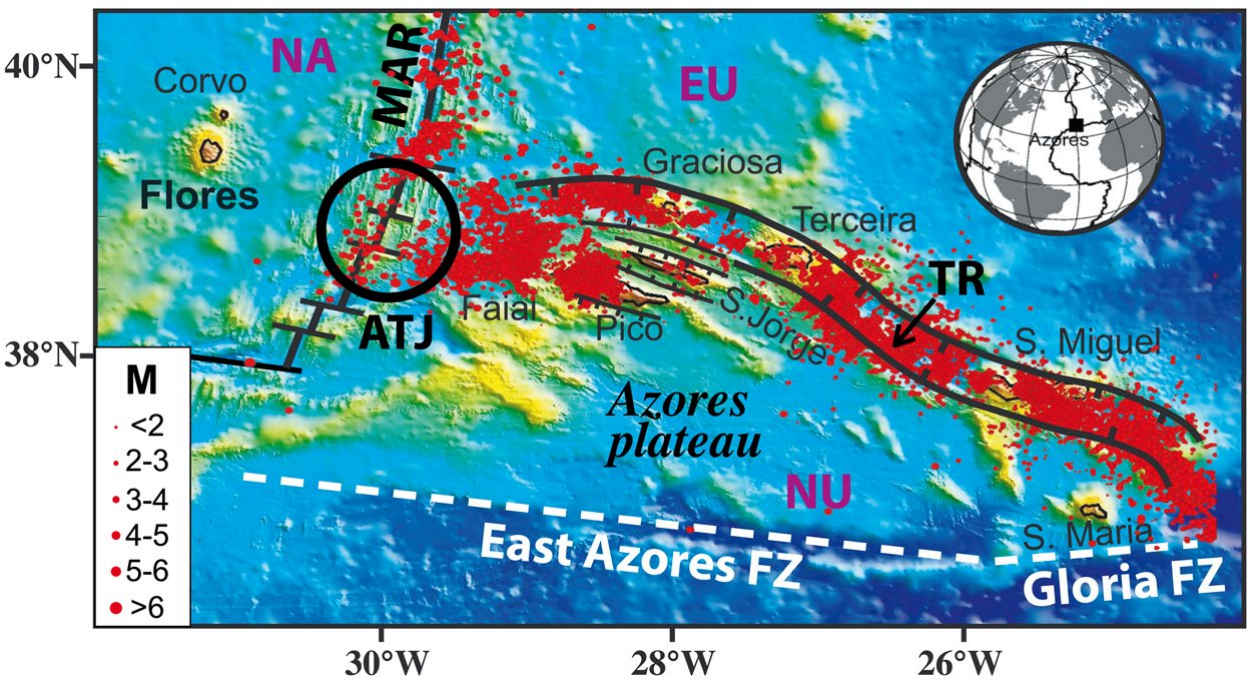
Location of Flores in the stable North American plate (NA). Black lines show the axis of the Mid-Atlantic Ridge (MAR) and associated fracture zones. Ticked lines bound the main horst and graben structures making up the present diffuse EU/NU plate boundary^34^. Background bathymetry from^69,70^. Seismicity over the period 1981–2011 (red dots) from CIVISA^71^; M: magnitude. TR: Terceira Rift, EAFZ: East Azores Fracture Zone.

Recently, LSMW with individual volumes up to a few tens of km^3^ have been recognized on small volcanic islands developed close to major plate boundaries, in either divergent or convergent settings, for instance in the eastern and central Azores (e.g.^18–24^), along the Caribbean arc (e.g.^25–28^), at Stromboli (e.g.^29,30^), or in Japan^31^. However, LSMW in those cases seems closely linked to active tectonics (e.g.^32,33^) and such islands thus may represent special cases in which the volcanic edifice would not fail in the absence of tectonic trigger. The intrinsic ability of small-size islands to experience LSMW thus remains a very controversial topic. Particular research efforts on this subject are needed to enable the elaboration of predictive models potentially applicable to volcanic edifices covering the full spectrum in terms of size, which ultimately has important implications for hazard assessment purposes. The main question we address here is therefore: Are small volcanic islands capable of undergoing LSMW even in the absence of triggering mechanisms like active tectonic faults and medium/high magnitude earthquakes? To answer this question, we focused on Flores (Azores), a small volcanic edifice developed in a stable tectonic setting. The island is located ca. 100 km west of the Azores Triple Junction (ATJ) where the American, Eurasian and Nubian plates meet (Fig. 1).The eastern branch of the ATJ is tectonically active, as marked by volcano-tectonic lineaments and recurrent regional earthquakes distributed along a series of horsts and grabens making up the present Eu-Nu plate boundary (e.g.^34–37^). In contrast, Flores Island sits on the western flank of the Mid-Atlantic Rige (MAR) in a zone with no historical seismicity (Fig. 1). Along with the small Corvo Island, Flores constitutes the emerged part of a N-S volcanic ridge roughly parallel to the adjacent portion of the MAR axis. Flores has a small size, with an area of ca 142 km^2^ and a total height above seafloor around 2400 m^17^. From previous studies, the island comprises several volcanic complexes mostly built on top of each other^38^ and has been active over the last 2 Ma, with dominant volcanic construction during the last 1 Ma^39,40^.

From new geomorphological, geological, geochronological and available bathymetric data, we show that the island is topographically and stratigraphically asymmetric, from which we infer that LSMW has removed at least half of the original volcanic edifice.

## Results

### Flores: a strongly asymmetric island

**Figure 2:**
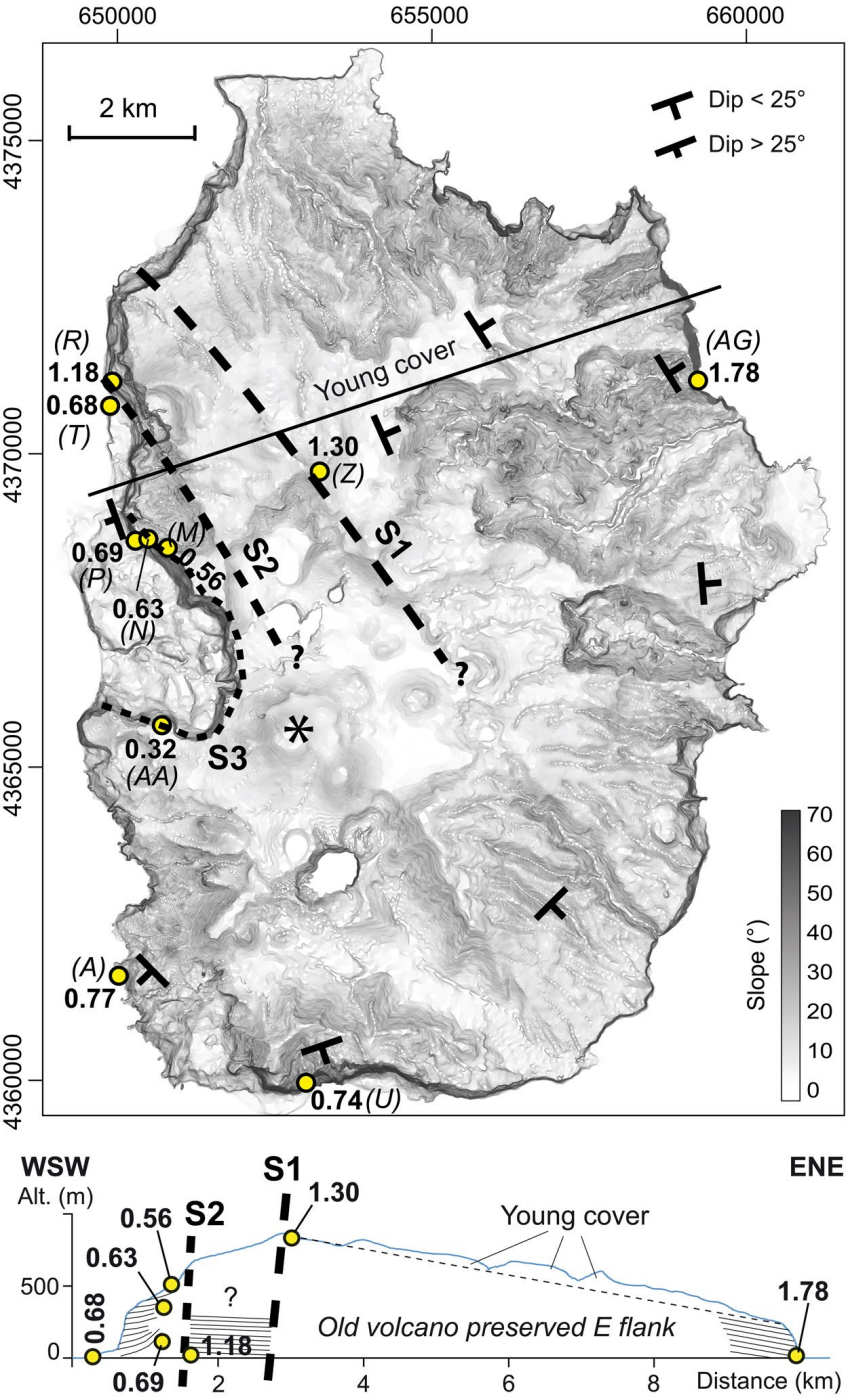
Gradient map generated from the DEM of the island (UTM projection, zone 25N, datum WGS84). The trace of the inferred scars is drawn with black dashed lines. Black star shows a recent volcanic edifice built along the trace of S2. The geometry of the various units and the new K/Ar ages are shown on the synthetic cross-section (position of profile indicated as black solid line on the map).

A gradient map complemented by an E-W topographic cross-section shows that the morphology of the island is strongly asymmetric (Fig. 2, Supplementary Fig. 1). Whereas the eastern flank exhibits a relatively regular slope locally deeply dissected by canyons, the whole western flank is truncated by steep breaks in slope. The main breaks in slope occur across two main NW-SE lineaments slightly concave to the west here interpreted as scars (S1 and S2 in Fig. 2). Their possible prolongation towards the SE is difficult to assess, as recent volcanic units have covered most of the central part of the island. An additional smaller but prominent horse shoe-shaped depression (S3 in Fig. 2, see also Supplementary Fig. 2, panel a) is visible on the western coast. It has been attributed to a slump^41^, the age and exact extent of which remain unknown.

**Figure 3:**
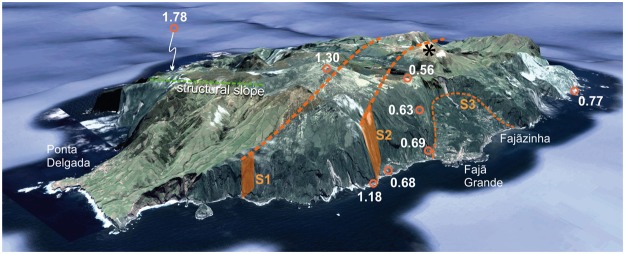
3D-view (Google Earth, © 2018 Google, Image © 2018 DigitalGlobe, Data SIO, NOAA, U.S. Navy, NGA, GEBCO) towards the SE, showing the relationships between the main units on the western flank of Flores. Our new K/Ar ages are shown in white. Recent volcanic edifice shown with a black star, as in Fig. 2.

New geological investigations complemented by K/Ar dating on separated groundmass (see Methods section) were conducted to identify the relationships between the main geological units and discuss the significance of the island-scale main E-W topographic and stratigraphic asymmetry. The eastern part of the island comprises an old volcanic succession with an overall eastward dip (Fig. 2). We sampled a lava flow from its base (sample FLO15AG) and obtained an age of 1.78 ± 0.03 Ma (Table 1, Fig. 2). In the upper part of the regular eastern slope, a lava flow near the summit of the island was sampled (FLO15Z) and dated here at 1.30 ± 0.02 Ma (Fig. 2). In contrast, volcanic units exposed on the western side of the island are systematically younger (Figs 2 and 3), which is in apparent contradiction with the overall eastward dip of the lava flows. The volcanic succession exposed at the foot of S1 comprises many flows cumulatively representing a thickness of several hundreds of meters. We sampled a sub-horizontal basal lava flow (FLO15R) from this succession at the shore level and dated it at 1.18 ± 0.05 Ma. This thick succession is cut by S2 and bordered immediately to the south by a few flows including local strombolian deposits exposed along the shoreline. We sampled the lowermost accessible lava flow (FLO15T) and dated it at 682 ± 13 ka (Fig. 2, Supplementary 2 panel a). Further inland, S2 is partially covered by a succession of thick lava flows exposed in a well-developed cliff, which we investigated along a pedestrian trail from near sea level to ca. 500 m altitude (Supplementary Fig. 2, panel a). Some of the lowermost lava flows have a steep westward dip, up to 40° (Figs 2 and 3), which implies that the lavas flowed over a steep topography, here interpreted as the continuation of S2 to the SSE. One of the basal lava flows was collected (sample FLO15P) and yielded an age of 693 ± 10 ka, which is undistinguishable within uncertainties from the age obtained on our sample FLO15T (682 ± 13 ka). Further up on the trail, two lava flow samples collected in the middle (FLO15N) and upper parts (FLO15M) of the same succession are here dated at 632 ± 9 ka and 558 ± 8 ka, respectively, in full agreement with the stratigraphic position (Fig. 2, Supplementary Fig. 2). Volcanic units cropping further south were also investigated, in order to assess their eventual relationship with the inferred discontinuities (Fig. 2). These include a lava flow on the western shore (sample FLO15A) intruded by many dykes and covered in unconformity by more recent units and a basal lava flow from a thick succession exposed along the southwestern cliff (sample FLO15U, Supplementary 2, panel b). These samples are here dated at 766 ± 12 ka and 742 ± 14 ka, respectively. These ages and their distribution on the island show that the western flank of the old volcano (>1.3 Ma) is missing and that the volcanic units exposed at the foot of the inferred prolongation of S2 are younger than ca 0.8 Ma throughout the whole western side of the island (Figs 2 and 3 and Supplementary Fig. 2).

**Table 1:** New K/Ar dating on separated groundmass (125–250 μm).

Sample	UTM 25E	UTM N	Altitude (m)	K%	^40^Ar* (%)	^40^Ar* (10^12^ at/g)	Age (Ma)	Unc. (Ma)
FLO15AG	659239	4371098	2	2.019	31.1	3.775	1.79	0.03
					34.4	3.753	1.78	0.03
						**mean**	**1.78**	**0.03**
FLO15Z	653043	4369748	905	3.134	40.7	4.236	1.29	0.02
					35.7	4.276	1.31	0.02
						**mean**	**1.30**	**0.02**
FLO15R	649883	4370943	2	1.457	2.7	1.808	1.19	0.05
					2.7	1.772	1.16	0.05
						**mean**	**1.18**	**0.05**
FLO15A	649983	4361747	3	3.407	16.1	2.729	0.767	0.012
					16.2	2.727	0.766	0.012
						**mean**	**0.766**	**0.012**
FLO15U	653101	4359882	20	1.440	8.3	1.117	0.743	0.014
					7.2	1.115	0.742	0.015
						**mean**	**0.742**	**0.014**
FLO15P	650283	4368655	176	1.470	24.3	1.069	0.696	0.010
					22.2	1.060	0.690	0.010
						**mean**	**0.693**	**0.010**
FLO15T	649851	4370690	5	1.955	7.8	1.417	0.694	0.013
					8.7	1.372	0.672	0.012
						**mean**	**0.682**	**0.013**
FLO15N	650381	4368709	357	2.238	27.6	1.484	0.635	0.009
					30.0	1.472	0.629	0.009
						**mean**	**0.632**	**0.009**
FLO15M	650721	4368518	528	1.608	17.6	0.9482	0.564	0.009
					31.7	0.9302	0.554	0.008
						**mean**	**0.558**	**0.008**
FLO15AA	650669	4365860	257	1.010	1.0	0.3189	0.302	0.03
					0.9	0.3444	0.326	0.04
						**mean**	**0.314**	**0.03**

Unc.: Uncertainties quoted at the 1σ level. For each sample, the mean is obtained by weighing by the amount of radiogenic argon (^40^Ar* (%)).

Finally, a westward dipping lava flow from the upper part of the volcanic succession cut by S3 (sample FLO15AA) is here dated at 314 ± 30 ka, revealing the late development of this arcuate structure.

### Offshore evidence for LSMW

**Figure 4:**
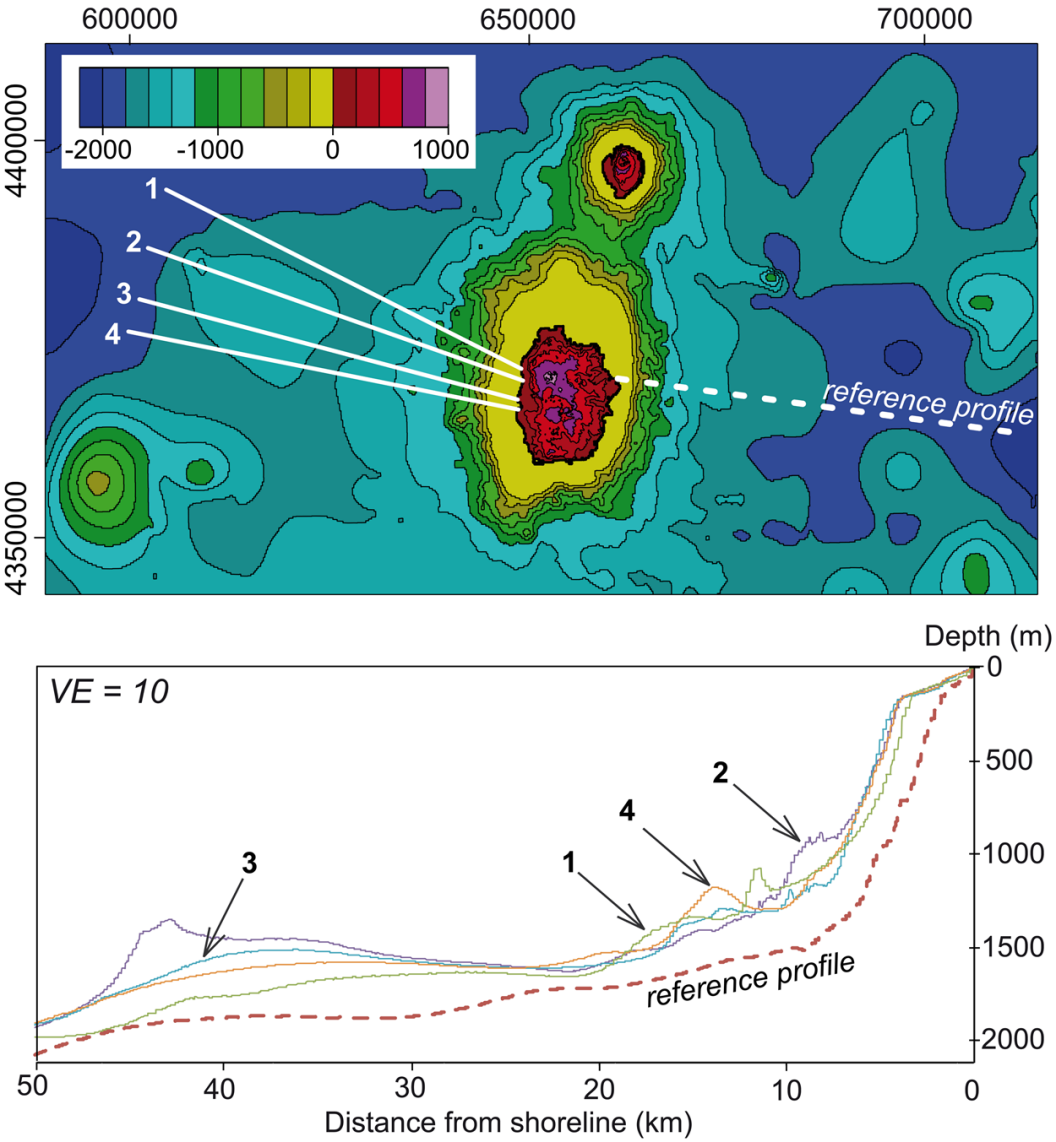
Upper panel: composite topography obtained by merging the onshore DEM with available bathymetric data around Flores (EMODnet portal). Coordinates in UTM (zone 25N). Color scale shows lines of iso-elevation (positive and negative values in meters above and below sea level, respectively). Lower panel: Bathymetric profiles across the eastern and western submarine slopes of Flores. Trace and name of profiles shown in upper panel. VE: vertical exaggeration. Note the great asymmetry between the western and eastern bathymetries, hundreds of meters higher in the west, where the bathymetry should be deeper if sediment/volcanic deposition were similar on both island flanks.

**Figure 5:**
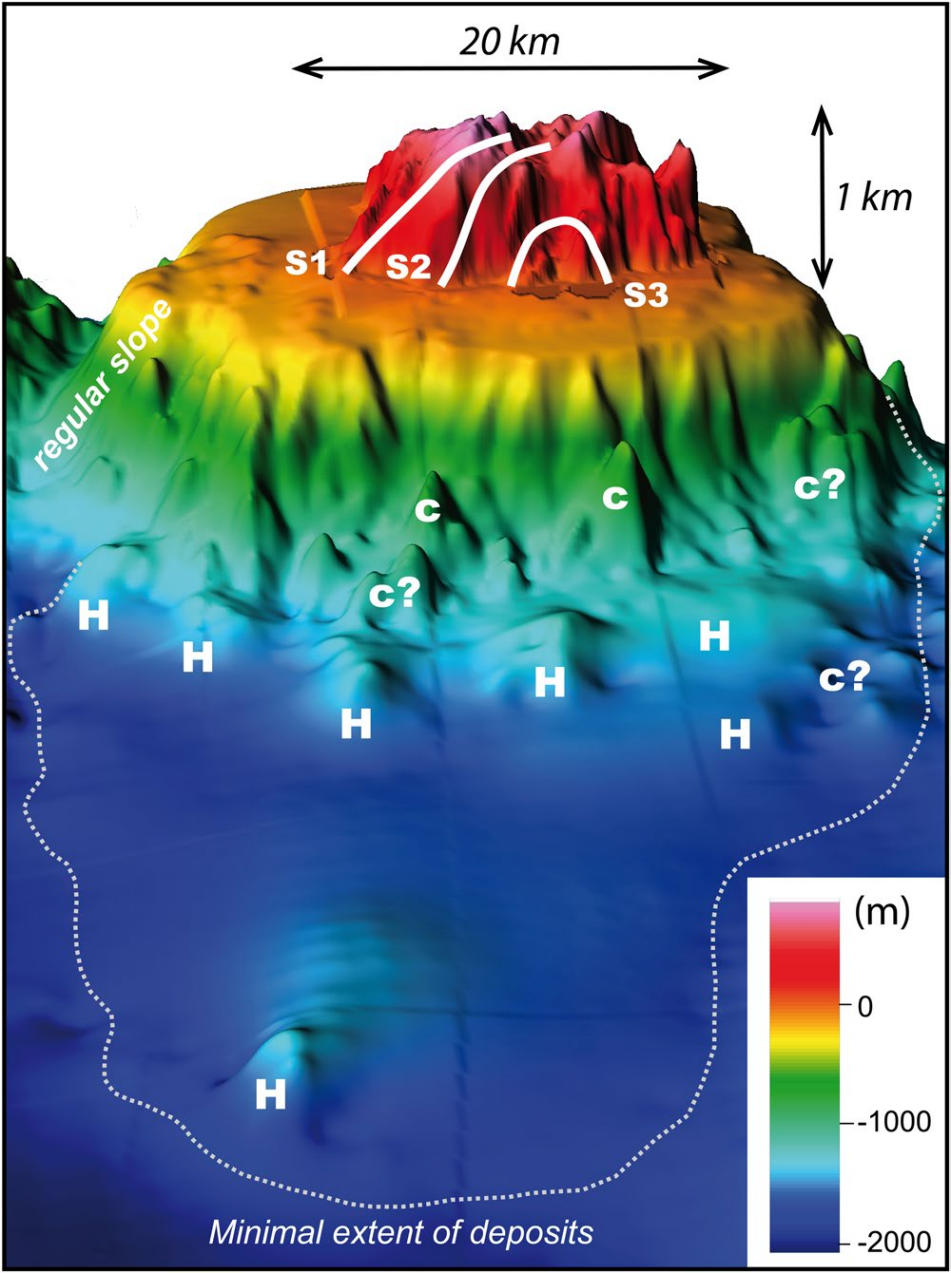
“Composite 3D-view towards the East, showing the on- and offshore characteristics of the western flank of Flores Island. Possible cones and hummocks are indicated with c and H, respectively.

**Figure 6:**
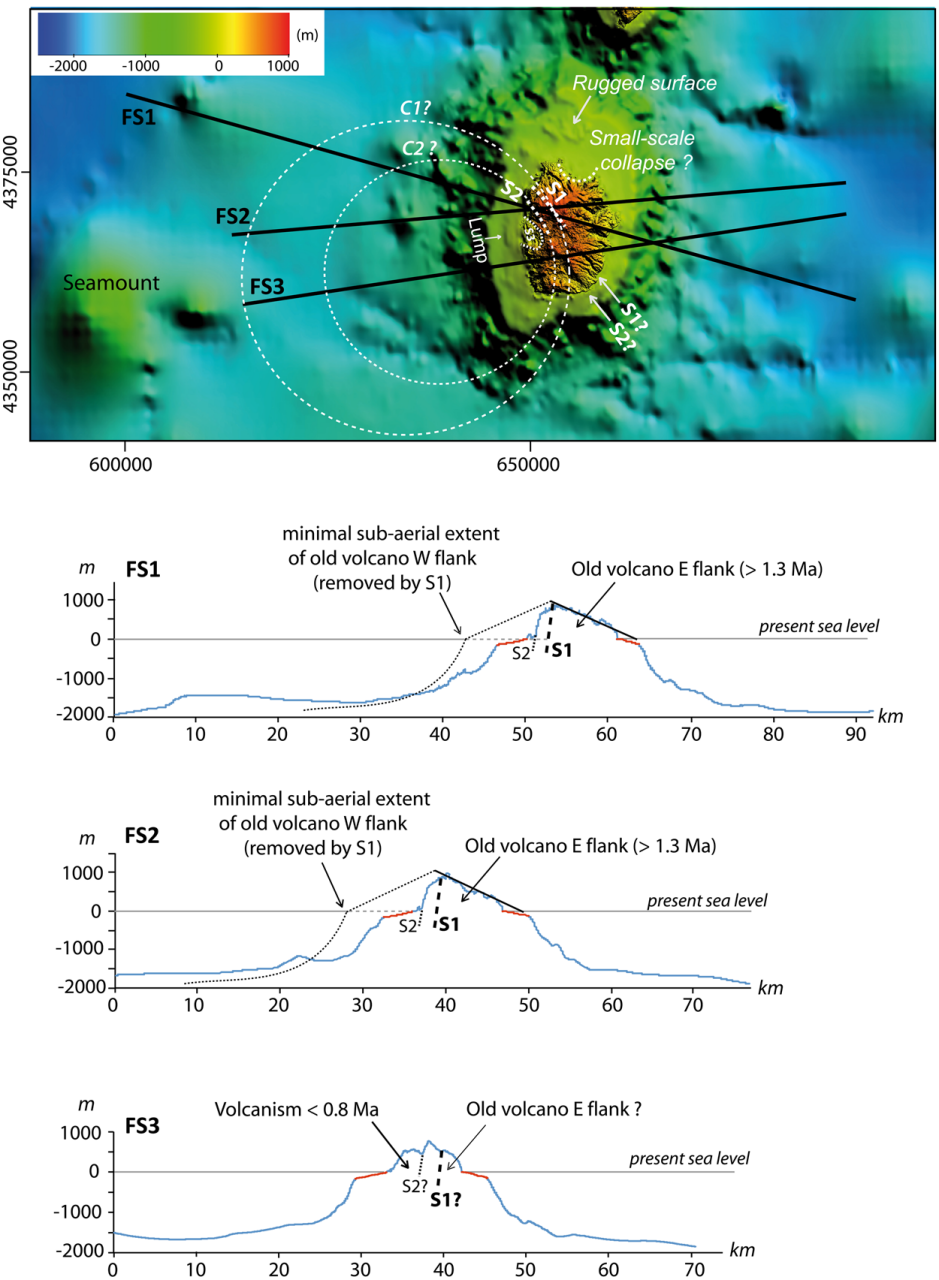
Full-sections (FS) across Flores Island and its submarine slopes. Coordinates on the map are UTM (zone 25 N); C1 and C2 refer to the eventual extent of sub-circular calderas, which would be much larger than the present island size, implying a former western flank even farther away to the west. In the cross-sections, dashed-lines show the minimal extent of the old volcano (**>**1.3 Ma), here inferred to have been initially symmetric and removed by large-scale, west-directed catastrophic flank collapse processes; submarine shelves shown with red lines.

From available marine data (EMODnet portal, see Methods section), the steep submarine flanks of the island extend down to ca. −1600 m (Fig. 4). The slope then rapidly becomes gentler down to −1800 m, which roughly coincides with the average depth of the seafloor in the area. The shallow submarine portion of the Flores volcanic edifice (depth < −200 m) comprises a smooth submarine shelf, which appears generally wider in the western sector (3-4 km) than in the east (2-3 km). The shelf is seemingly more developed in the North probably due to the proximity of Corvo shallow submarine slope (Figs 4 and 5), but its rugged topography (Fig. 6) additionally suggests the presence of shallow volcanic cones or small-scale landslide debris generated by recent northward small-scale collapse processes.

At the larger scale, bathymetric profiles across the western and eastern submarine slopes of the island highlight a strong topographic asymmetry (Fig. 4). The submarine western flank appears much shallower than the eastern flank by several hundreds of meters. This is in apparent contradiction with what would be expected if volcanic and sedimentary deposits were similar on both island flanks, as the oceanic crust generally gets older and deeper towards the west. The eastern submarine slope of Flores has an overall logarithmic profile typical of the un-failed constructional flanks of a volcanic island (e.g.^7^), which we therefore considered as a “reference profile” (Fig. 4). In comparison, four bathymetric profiles west of Flores highlight the presence of significant positive bathymetric anomalies, on the order of hundreds of meters, extending up to 50 km away from the island shore. A first anomaly is observed between 7 and 20 km, it reaches up to 500 m in magnitude and shows a highly irregular scattered surface (Fig. 4). Further away, a second main positive depth anomaly is visible between ca. 25 km and 45 km from the island shore. It appears relatively smoother, which may be due to the lower resolution of the bathymetric data in this distal portion. However, this second positive bathymetric anomaly is wider than the first one and is still prominent (ca. 500 m high).

A synthetic 3D perspective-view at the full scale of the Flores western flank (Fig. 5) provides additional insights. The possible offshore prolongation of S1 interrupts the northwestern regular submarine slope of the island and is immediately bounded by a prominent hummock with an irregular shape and a lateral width of ca. 2 km (Fig. 5).

Similar large irregular hummocks can be inferred over the distal western submarine flank, up to a distance of ca. 50 km, where a significant asymmetric relief could likely represent the upper part of a large un-fragmented slid block (Fig. 5 and Fig. 4, profile 2). Smaller and shallower mounds with a conical shape are observed on the proximal submarine slope of the island (distance from shoreline < 20 km). They appear well enclosed in the offshore prolongation of S2, the NW limit of which is marked by an abrupt narrowing of the insular shelf. These small-scale features could either represent landslide-related blocks or volcanic cones, but further evaluation would require higher-resolution bathymetric and/or seismic data such as the ones acquired recently in the eastern Azores Islands^42,43^.Finally, the lateral rims of the prominent arcuate scar S3, do not show major morphological imprint in the adjacent submarine shelf, which suggests that S3 is a very shallow structure. We note, however, that a subtle lump on the shelf can be suspected immediately offshore S3 (Fig. 6), which could reflect recent (active?) outward movement along a shallow (depth < 200 m) long-angle basal decollement.

## Discussion and Conclusions

Some NW-SE lineaments on Flores, including the structure here termed S2, have been previously interpreted and mapped as “fractures”^40^. From the linear to slightly concave character of these structures, one may propose that they represent west-dipping normal faults affecting the island and the underlying oceanic crust. However, such possibility appears unlikely, because the NW-SE fault trend is not known in the lithosphere where Flores sits. Moreover, the island has grown in a tectonic environment with no significant seismicity recorded between 1926 and 2015 (e.g.^44,45^). Regional seismic events over longer periods cannot be totally excluded, as earthquakes occasionally occur in intra-plate interiors, as reported for the Indian plate^46^. However, the Indian oceanic lithosphere is significantly affected by the Himalayan collision, which promotes large-amplitude deformation, a process not directly transposable to the study area. From half-spreading rates at the MAR in the sector^47,48^, Flores was located ca. 20 km farther east 2 Ma ago, in a tectonic setting similar to present. Therefore, major earthquakes (M > 6) able to trigger LSMW in Flores during the last 1.3 Ma appear unlikely.

The NW-SE direction here observed on Flores also departs from any structural direction known in the area, as the MAR fabric strikes NNE-SSW, whereas fracture zones offsetting individual MAR segments strike mostly WNW-ESE. None of these directions has been observed on Flores. In contrast, we here show that Flores is made up of successive nested volcanic units separated by major scars, which contributed to shape the island towards a strongly asymmetric volcanic edifice. One may argue that the scars here recognized could represent part of sub-circular central calderas filled by more recent lava units and then eroded preferentially in the west by marine erosion. However, from the shape of both S1 and S2 and our new onshore data, the dimensions of such calderas would exceed by far the present size of the island (Fig. 6), which seems unlikely for such a small edifice. The central caldera collapse hypothesis also does not account for the observed E-W asymmetry, i.e. it does not explain adequately why the whole (sub-aerial and submarine) western flank of the island is missing. Assuming a former symmetrical morphology along the E-W direction, the sub-aerial western flank of the main volcano (>1.3 Ma) had to reach sea level at least 8 km away from the present island shore (Fig. 6). This is twice the extent of the western submarine shelf. Therefore, removal of the western flank solely by marine erosion seems insufficient.

From the geometry of the contacts, the age and attitude of the volcanic successions and the presence of inferred debris deposits offshore (Figs 3–6), westward failure of the island flank therefore appears the most plausible scenario.

Two main types of large landslides are classically distinguished on volcanic islands^3^: (1) slow-moving slumps, which gradually displace the island flank along arcuate structures eventually connected to a deep basal decollement, at rates of a few mm/yr to a few cm/yr^4,18^, as the Hilina Slump in Hawaii; and (2) catastrophic landslides, which rapidly remove part of the island flank and produce fast-running debris-avalanches with potentially hazardous consequences regarding tsunami triggering (e.g.^2,13^). The two mechanisms are not mutually exclusive, as slow-moving landslides can experience significant acceleration and suddenly evolve into a catastrophic failure (e.g.^49^). On Flores, small-scale slumping processes have been proposed^41^ and could explain the existence of the arcuate S3 structure. One may argue that this small structure is just part of a much larger slump complex, i.e., that S1, S2 and S3 may have been active at the same time, gradually displacing the western flank of Flores throughout the last 1.7 Ma. However, this is not supported by the new ages reported here. The base of the thick succession at the foot of S1, here dated at 1.18 ± 0.05 Ma, is significantly younger than the topmost flows of the eastern preserved flank (1.30 ± 0.02 Ma). Therefore, the most plausible explanation is that failure on S1 removed, after 1.3 Ma, the whole western flank of Flores in a catastrophic way, so producing a long-running debris avalanche as inferred from the large positive bathymetric anomaly west of Flores and the presence of large kilometric blocks with irregular polygonal shape at a distance exceeding 25 km from the present island shore (Figs 4 and 5). The landslide depression was then buried from 1.18 Ma onwards by subsequent volcanic activity, as observed on many larger volcanic islands, where massive catastrophic flank collapses have been immediately followed by the rapid growth of a new volcano inside the landslide depression (e.g.^11,15,50–54^). From our new data, the first west-directed landslide (S1 scar) thus occurred between 1.30 Ma and 1.18 Ma.

The arcuate S2 scar cuts the succession younger than 1.18 ± 0.05 Ma and is covered in unconformity by lava flows from the volcanic successions here dated between ca 770 ka and 350 ka. This yields a younger temporal bound for the second flank collapse (S2), which is therefore constrained between ca. 1.18 Ma and 0.80 Ma. From their dip, the younger flows (<800 ka) were erupted either from a main single volcano located near the central western half of the island and/or from a series of small volcanic edifices distributed along the rim of S2 (Figs 2 and 3, Supplementary Fig. 1). Some of these young flows may have cascaded onto the steep S2 headwall and buried the landslide depression, getting closer to horizontal near sea level as they covered the inferred debris (Fig. 2). The overall development of the young volcanism preferentially to the west can reasonably explain the absence of an obvious large embayment in the mid-submarine slope of Flores, in contrast with the El Golfo slide in El Hierro^9^, which is very young and has not yet been covered by significant volcanism. The absence of obvious major submarine scar is also typical of many other landslides older than 500 ka, e.g. on the western flank of La Palma^10^, or to the North of Tahiti where post-landslide volcanic units extensively concealed the collapse scar and buried the proximal submarine debris-avalanche deposits, down to ca. 1000 m below current sea level^7^.

Robust quantification of volumes involved in the successive failures of Flores western flank is not directly possible from the available data, as the 3-D geometry of the various structures and the extent of the offshore deposits is not precisely known. However, our data suggest that the whole western flank of the island has been suddenly removed at least once during a major geological event (oldest landslide, S1 scar), possibly involving successive multi-stage retrogressive failures, as proposed on Tenerife Island in the Canary^13,55^. On Flores sections, the sub-aerial missing flank west of S1 can be roughly assimilated to a triangle with a height and a width of 1 km and 8 km, respectively (FS1 and FS2, Fig. 6). The corresponding volume can thus be estimated from a wedge, with a minimal length of ca 10 km along the N-S direction (northern half of the island). The corresponding sub-aerial missing volume reaches 40 km^3^. Taking into account the possible extension of the old volcano in the South (profile FS3, Fig. 6) and the submarine portion west of the island, the total volume removed may exceed 100 km^3^, which is on the same order as the volume removed by some large-scale landslides on much larger intra-plate volcanic islands, e.g. in the Canaries and in Cape Verde (see updated database in Costa *et al*.^20^). The estimated run-out for the distal debris offshore Flores (ca. 50 km) is also similar to the values observed for the Las Playas landslide in El Hierro (Canary), the Güimar landslide in Tenerife (Canary), the Monte Amarelo landslide in Fogo (Cap Verde) and the Top de Coroa landslides in Santo Antão (Cape Verde), despite the much smaller vertical drop for Flores (H < 2.5 km vs H > 4 km for all the cases mentioned above). This indicates that the slides here reported were not slowly moving (slump) and supports effective friction reduction and high velocity of the debris at the time of sliding (e.g.^56,57^), which has potential important implications for tsunami generation. Therefore, we infer that small-size volcanic islands like Flores (total height above seafloor around 2500 m or less) can actually experience LSMW (>40 km^3^) with potentially catastrophic consequences. The systematic search for large landslides on similar volcanic islands and seamounts thus constitutes an important challenge for the future, as (1) many small oceanic volcanic edifices, similar to Flores, are scattered in the various oceans and seas all over the globe, so most likely greatly increasing the currently estimated landslide frequency and therefore the risk; and (2) tsunamis potentially generated by large flank failures such as the ones here reported may travel long distances and impact the surrounding shorelines, thereby constituting a ubiquitous potential threat.

## Methods

### Geomorphological analyses

The first-order topographical characteristics of Flores Island were derived from a high-resolution digital elevation model (DEM). The digital elevation model was supplied by the Regional Government of Azores (Direção de Serviços de Planeamento e Gestão de Meios) and was produced by the Army Geoespatial Information Center (CiGEoE). The elevation model was computed using a set of contour lines, spot heights and breaklines acquired from aerial stereo-restitution. The aerial photos were acquired at 1:22500 scale. The original data provided by CiGeoE has a grid node spacing of 5 m. These data were used to generate shaded-relief maps with different illuminations, a gradient-map (illumination from the top) and topographic profiles to visualize the main volcanic units and the scars highlighted by steep slopes (>50°). A 3D view of the island was additionally realized (Fig. 3) to illustrate the geometric relationships between the main units. The main morphological characteristics of Flores submarine flanks were additionally investigated from bathymetric data available from the EMODnet portal^58^ (http://portal.emodnet-bathymetry.eu). The DEM is based on data provided by the IHPT hydrographic Institute (Portugal), complemented by a few multi-beam bathymetric lines (EMEPC, Portugal) and integration of the GEBCO digital bathymetry for gaps with no data coverage. The bathymetric grid, which has an X-Y spacing varying from ca 100*100 m in the proximal offshore domain to 230*230 m in the distal western sector, is useful for analyzing the first-order main morphological features. Bathymetric profiles were then realized to compare the morphology of the various sectors. We focused especially on the E and W submarine flanks, which are not disturbed by secondary volcanic features, i.e., the submarine flanks of Corvo Island in the North and a large seamount in the Southwest. Finally, the on-land and offshore data were merged to produce a 3-dimensionnal view allowing visualization of the main topographic characteristics at the full scale of the volcanic edifice (Figs 5 and 6).

### Geological investigations and sampling

Fieldwork was aimed at recognizing the main geological units and their geometric relationships. Observation of the various volcanic successions was achieved by boat and on-land through all accessible outcrops, especially along road cuts, canyons and cliffs, which provide maximum exposure. Particular attention was paid to the general dip of the main volcanic successions to infer the location and the overall morphology of the initial volcanoes. This was achieved by measuring the main attitude of the flows along intersecting planes with variable orientation in order to get the true dip, whenever possible. Further examination of the main unconformities separating lava piles was achieved to document the different destruction processes occurring at various scales (tectonics, erosion and large-scale mass-wasting).

Sampling was achieved preferentially on the base and the top of individual volcanic successions to characterize the age and duration of the main volcanic episodes and to constrain the age and functioning of the various structures. Sampling was systematically achieved on well-identified lava flows, attested by the presence of scoriaceous base and top (klinker). The central massive part of the lava was extracted by means of hammers and chisels and broken *in-situ* with a sledge-hammer to reach the freshest, bubble-free core of the flow and reject any vesicle-rich part. Careful examination of the rock in the field was realized to discard samples showing obvious traces of weathering.

### K/Ar dating on fresh separated groundmass

Thin-sections were systematically realized to characterize the texture, cristallinity and freshness of samples. Most of them are basic to slightly evolved in composition, so the volcanic groundmass was chosen for subsequent geochronological analyses. After crushing and sieving at an adequate fraction (typically 125–250 μm), samples were washed in dilute nitric acid and rinced with deionized water. Magnetic separator and heavy liquids were systematically used to eliminate phenocrysts (olivine, pyroxene, plagioclase), which may carry unsuitable excess-argon and to extract the unaltered and homogeneous fraction of the groundmass in order to get a meaningful eruption age.

The unspiked K/Ar Cassignol–Gillot technique^59^ allows the precise determination of small amounts of radiogenic argon (^40^Ar*) and is particularly appropriate to date late quaternary basalts and andesites with low-K contents with an uncertainty of a few ka (e.g.^6,15,60–62^). The technique has even been extended to the last millennium with an uncertainty of only a few centuries in the case of high-K lavas^63^. This technique has been shown especially suitable to constrain the timing of large flank failures on other volcanic islands (e.g.^6,11,12,27,51^).

The analytical data and our new K/Ar ages are presented in Table 1 and shown in Figs 2 and 3. Decay constants of Steiger and Jäger^64^ have been used. Full details on the analytical procedure and performances are given elsewhere^59,65^. The relative uncertainty (σ_age_) on each individual age determination is obtained as follows:

$${\sigma }_{{\rm{age}}}=\sqrt{{(\sigma {\rm{K}})}^{2}+{(\sigma cal)}^{2}+{(\sigma A{r}^{\ast })}^{2}},$$

from the quadratic sum of all 3 independent sources of uncertainty involved in the calculation: (1) the relative uncertainty on the K-content determination (*σ*_K_), (2) the relative uncertainty on the calibration of the ^40^Ar signal (*σ*_*cal*_) and (3) the relative uncertainty on the correction of the atmospheric contamination (*σ*_Ar*_ = 0.1/^40^Ar* × 100), ^40^Ar* being the radiogenic content.

1. The relative uncertainty on K-content measurement is about 1%, from repeated measurements on standards MDO-G and ISH-G^66^ and BCR-2^67^. For a given sample, repeated K measurements (usually twice) on distinct aliquots are achieved until reaching an average value with a relative standard deviation (RSD) better than 1%.
2. The calibration of the ^40^Ar signal on our mass-spectrometer is obtained by systematic measurements of an air pipette, which is routinely compared to the GL-O standard with its recommended value of 6.679 × 10^13^ at.g^−1^ of radiogenic ^40^Ar^68^ and regularly checked using analyses on other standards, e.g., MMhb-1 and HD-B1 standards (see Germa *et al*.^60^ for more details). The relative uncertainty on the calibration, including the standard uncertainty, is 1%.
3. The radiogenic argon content (%^40^Ar*) is measured by comparison of the ^40^Ar/^36^Ar ratio of the sample with an air-pipette, measured under strictly similar Ar pressure conditions. This can be achieved because of the very stable analytic conditions of our mass spectrometer. The limit of detection of the radiogenic Ar content is presently of 0.1%, which allows ages as young as 2 ka to be obtained with only a few centuries uncertainty in favorable cases^65^.

For samples with low radiogenic yield (<10%), the uncertainty on the age is dominated by the uncertainty on the atmospheric contamination and can reach several tens of %. For ^40^Ar* higher than 10%, the uncertainty on the correction of atmospheric contamination becomes negligible and the total age uncertainty rapidly converges towards 1.4% (i.e. $\sqrt{{(1)}^{2}+{(1)}^{2}}$). When the individual ages overlap within their range of uncertainties, the mean age is calculated by weighting each individual age with the amount of radiogenic argon. Note that this method is somehow conservative, but it prevents us from underestimating the systematic uncertainties.

## Electronic supplementary material

Supplementary information
