# Supplementary material for: Large-scale mass wasting on small volcanic islands revealed by the study of Flores Island (Azores)
Source: Sci Rep. 2018 Sep 17;8:13898. doi: 10.1038/s41598-018-32253-0 (PMC6141455; doi:10.1038/s41598-018-32253-0)

Large-scale mass wasting on small volcanic islands revealed by the study of Flores Island (Azores)

A. Hildenbrand[1*], F.O. Marques[2], and J. Catalão[3]
[1] GEOPS, Univ. Paris-Sud, CNRS, Université Paris-Saclay, 91405 Orsay, France.
[2] Universidade de Lisboa, Lisboa, Portugal.
[3] Instituto Dom Luiz (IDL), Faculdade de Ciências, Universidade de Lisboa, Portugal.

*corresponding author: Email: anthony.hildenbrand@u-psud.fr

Supplementary Information

Supplementary Figure 1: Clean version of Figure 2, without interpretation.

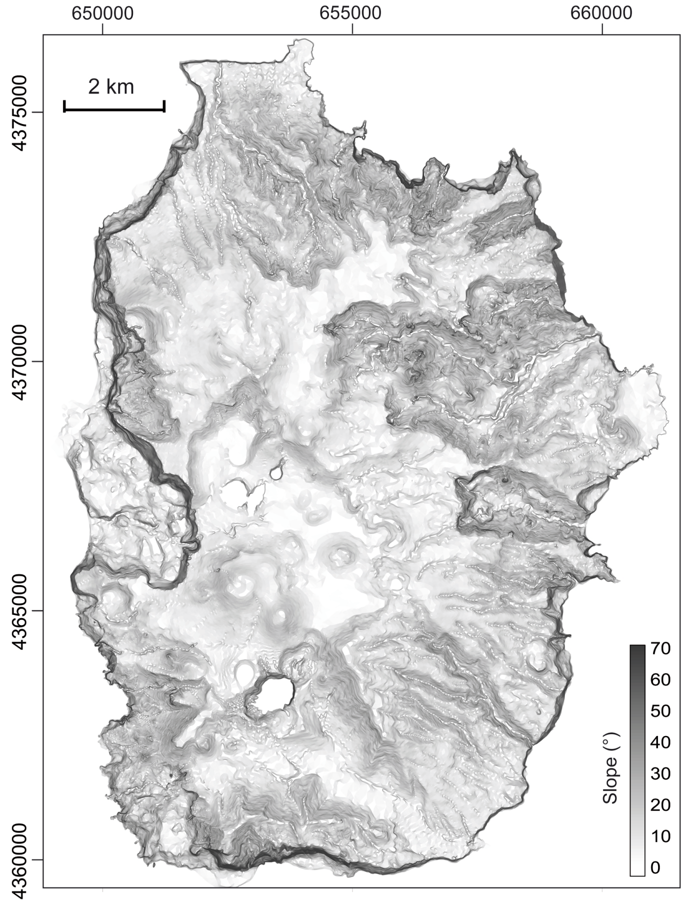

Supplementary Figure 2: Photographs (taken by AH) of the main units and scars on the western flank of Flores. The camera symbol on the inset map shows the point of view from which the pictures were taken.

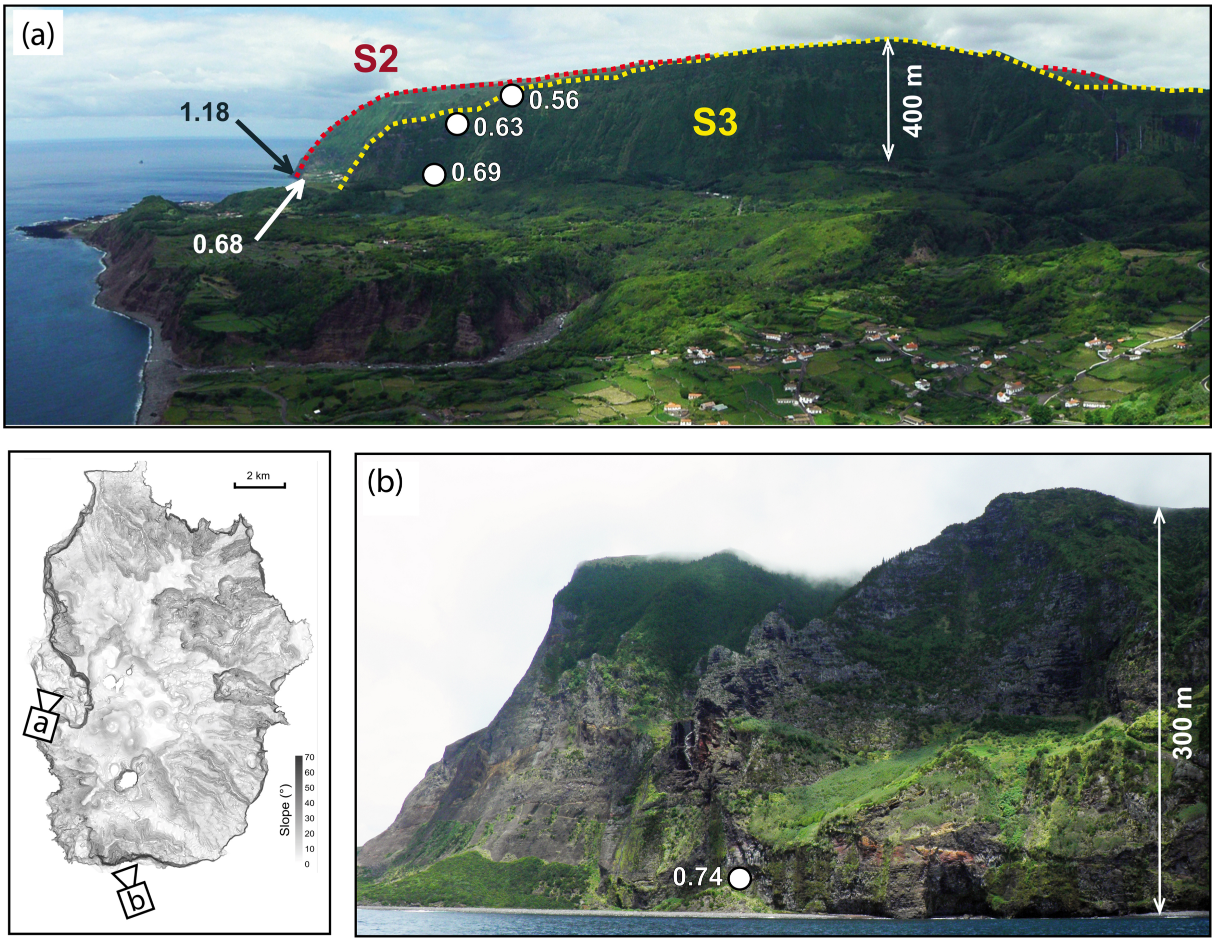

Supplement: Supplementary file 1 — Supplementary information [file 41598_2018_32253_MOESM1_ESM.pdf]
